# Supplementary material for: Modulating splicing in 5’ untranslated regions to treat rare haploinsufficient disease
Source: bioRxiv. 2025 Dec 9:2025.12.07.692584. Preprint. [Version 1] doi: 10.64898/2025.12.07.692584 (PMC12822670; doi:10.64898/2025.12.07.692584)
Supplement: Supplementary file 1 [file NIHPP2025.12.07.692584v1-supplement-2.pdf]

bioRxiv preprint doi: https://doi.org/10.64898/2025.12.07.692584; this version posted December 9, 2025. The copyright holder for this preprint (which was not certified by peer review) is the author/funder, who has granted bioRxiv a license to display the preprint in perpetuity. It is made available under a CC-BY 4.0 International license.

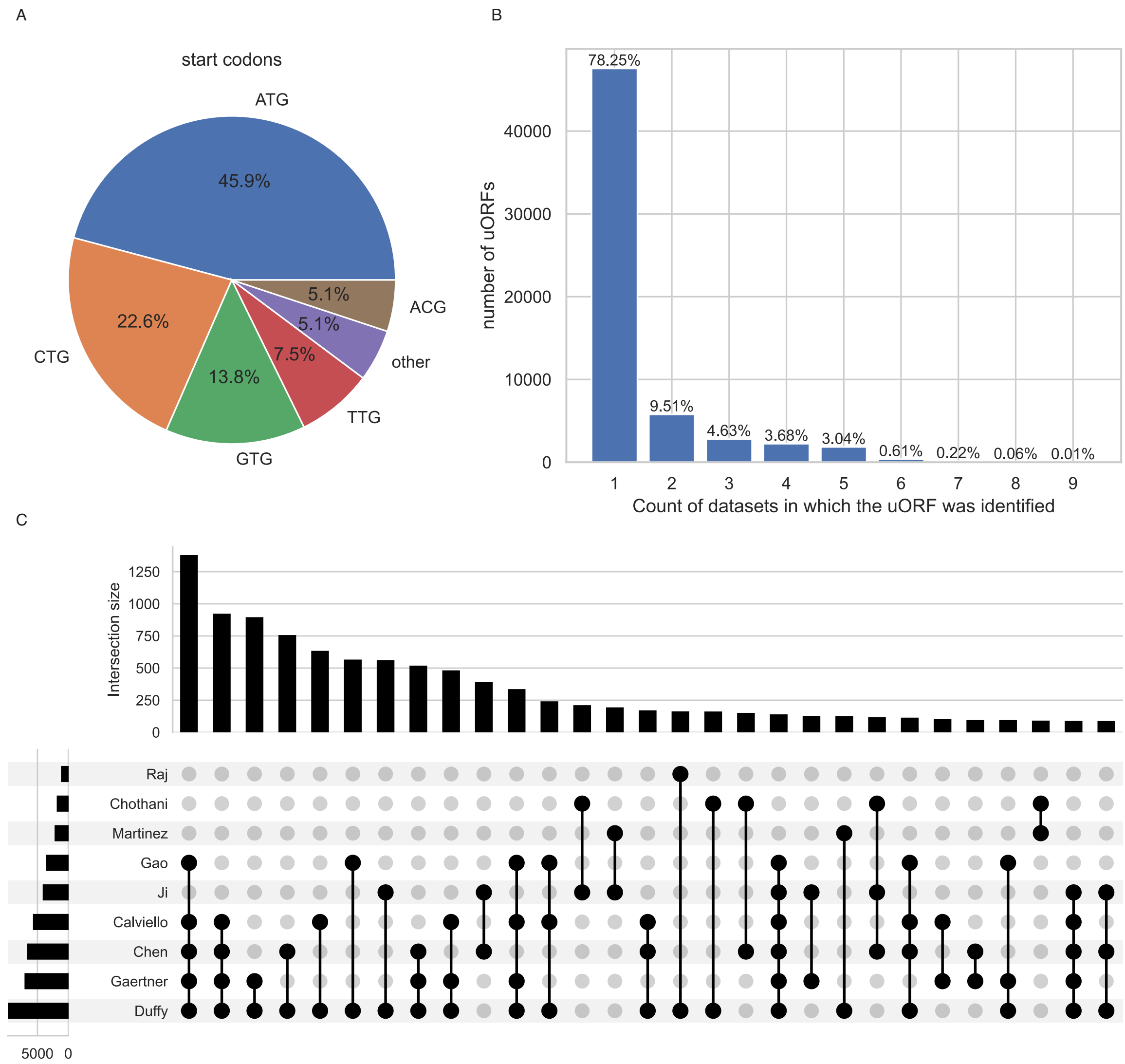

Supplementary Figure 1: Overview of the uORF data collated in this pipeline. A) Proportion of codon sequences identified as the start site of a uORF. **B)** The number of sources which identify each uORF. **C)** Intersection between the sources (minimum subset of uORFs = 80).

bioRxiv preprint doi: https://doi.org/10.64898/2025.12.07.692584; this version posted December 9, 2025. The copyright holder for this preprint (which was not certified by peer review) is the author/funder, who has granted bioRxiv a license to display the preprint in perpetuity. It is made available under a CC-BY 4.0 International license.

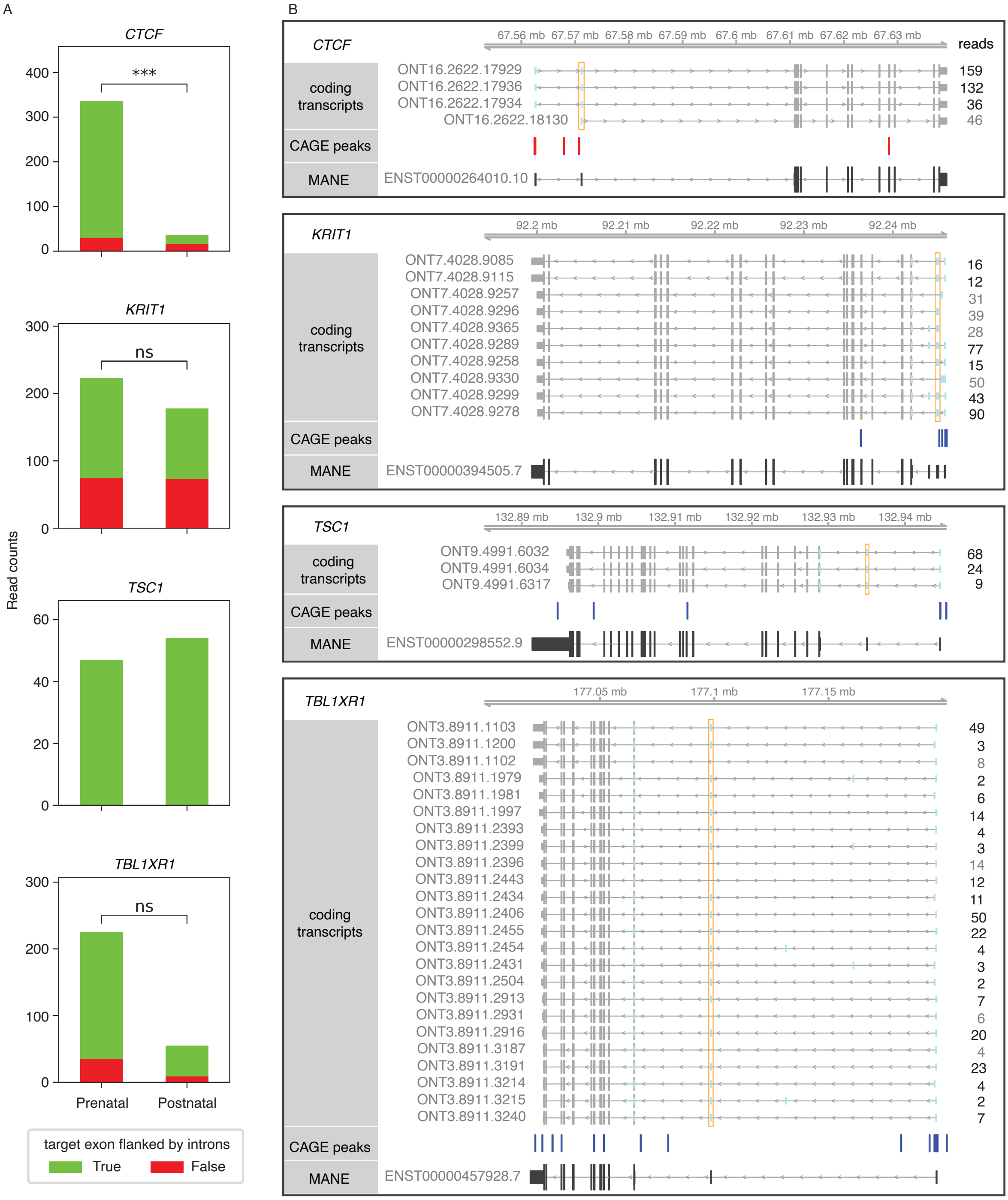

Supplementary Figure 2: For selected genes **A)** the number of reads from transcripts categorised by whether the target exon is skippable, flanked by introns, in prenatal and postnatal samples. **B)** Isoforms identified by long-read sequencing, predicted to be coding[27], and supported with a CAGE peak. For *CTCF* only there was a significant decrease in the proportion of reads from transcripts where the target exon can be skipped between prenatal and postnatal samples (fisher exact test, odds ratio = 0.11, $p < 0.001$).

bioRxiv preprint doi: https://doi.org/10.64898/2025.12.07.692584; this version posted December 9, 2025. The copyright holder for this preprint (which was not certified by peer review) is the author/funder, who has granted bioRxiv a license to display the preprint in perpetuity. It is made available under a CC-BY 4.0 International license.

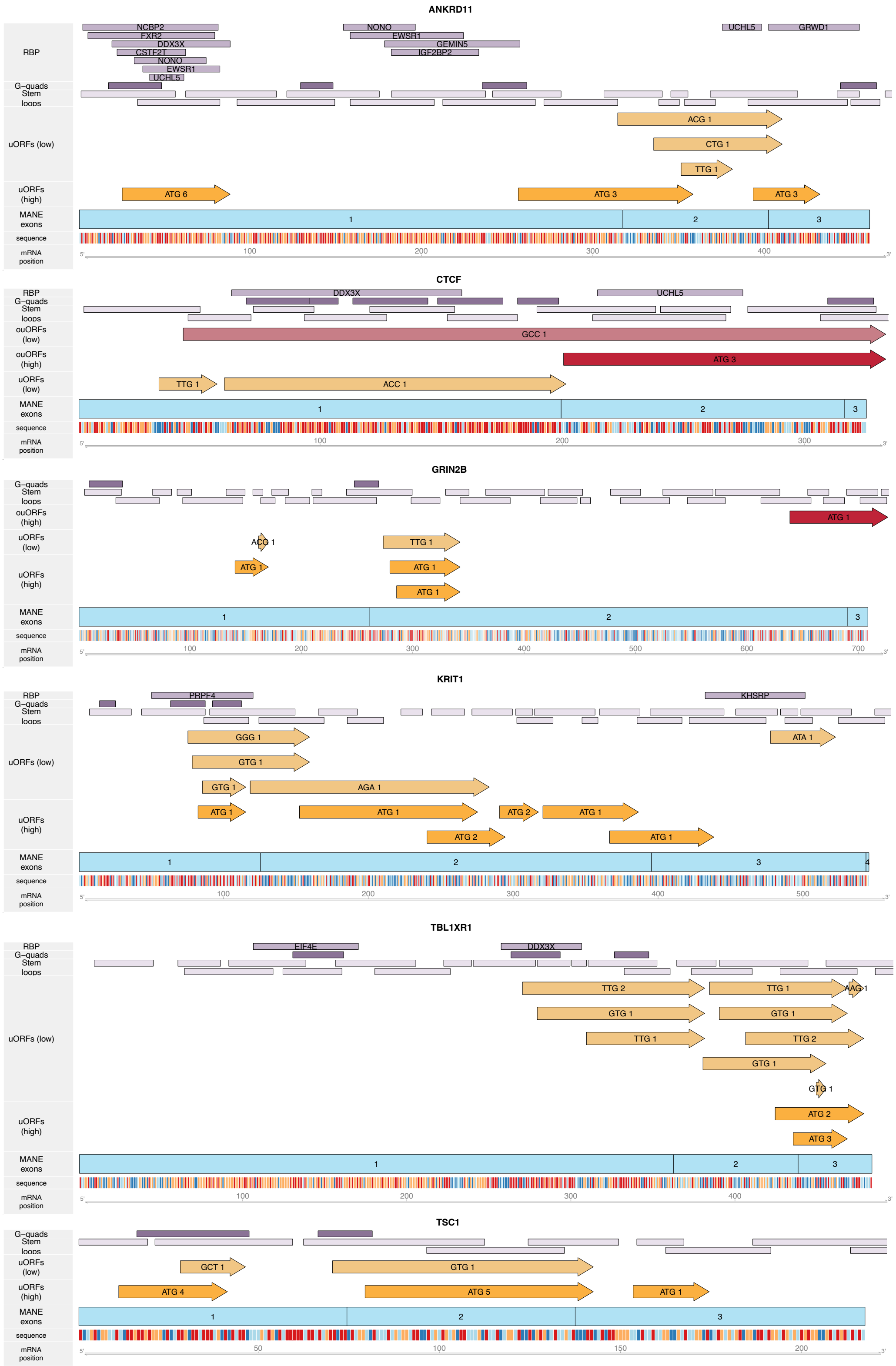

Supplementary Figure 3: Schematics of 5’UTR features in prioritised gene targets. 5UTR exons are light blue, uORFs are annotated in orange, ouORFs are annotated in red, start codons are highlighted along the sequence. G-quadruplex sections, stem loops and RBPs are annotated in purple-grey. There are no known IRES sites in these six target genes.

bioRxiv preprint doi: https://doi.org/10.64898/2025.12.07.692584; this version posted December 9, 2025. The copyright holder for this preprint (which was not certified by peer review) is the author/funder, who has granted bioRxiv a license to display the preprint in perpetuity. It is made available under a CC-BY 4.0 International license.

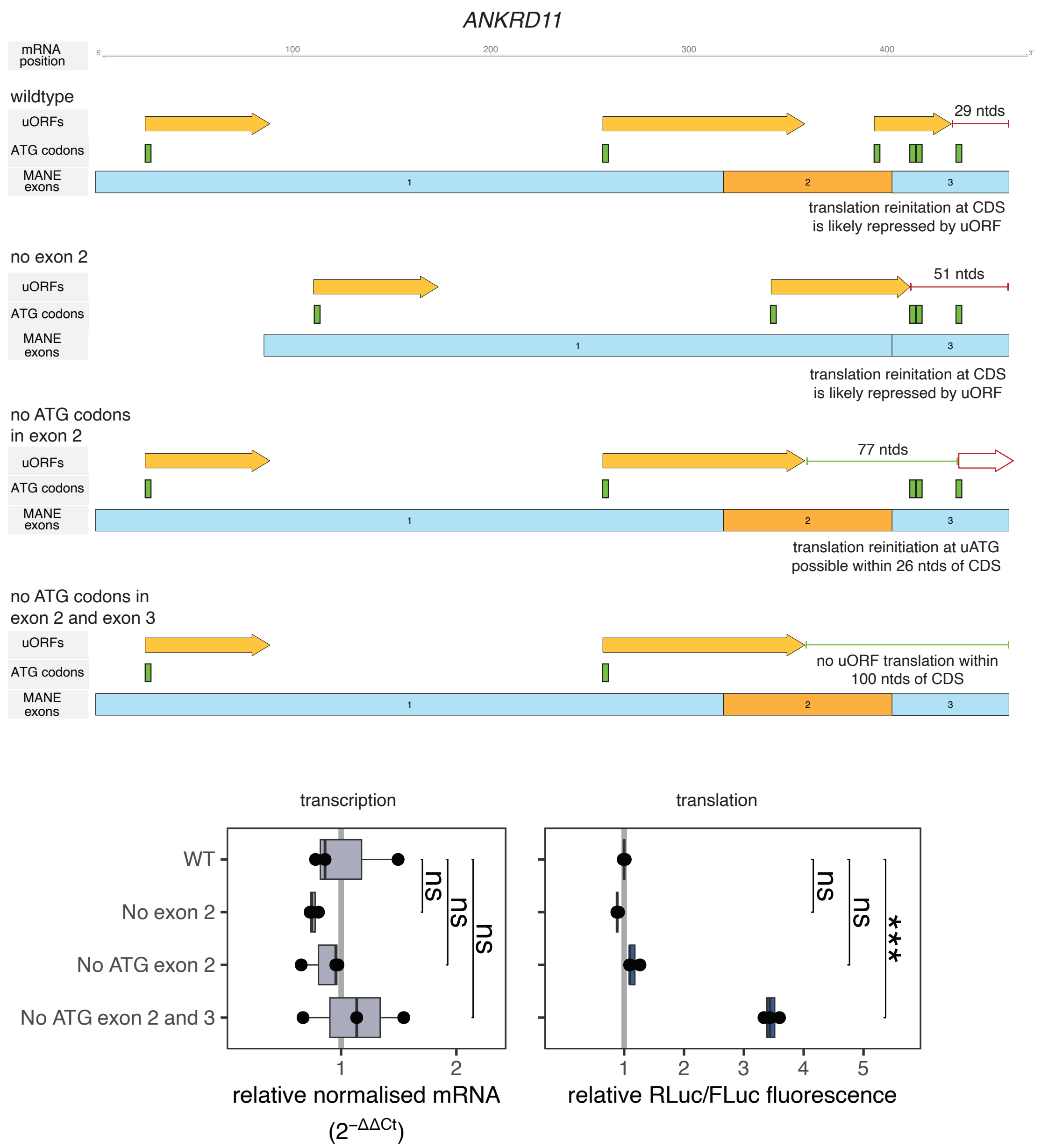

Supplementary Figure 4: Redundancy between uORFs and normally untranslated uATGs in *ANKRD11* and the impact on exon skipping. In *ANKRD11* the uORF initiating in the target exon has an intercistronic region of 29 ntds which is unlikely to facilitate translation reinitiation at the CDS. However, when the target exon is skipped the uORF initiating in exon 1 extends, such that there is only a marginal increase in the intercistronic region. Further, when the ATG site is mutated, reinitiation may occur at ATGs within exon 3. It is only when the uORF in exon 2, and the ATG codons downstream are removed that translation from the CDS increases.
